# Supplementary figures and images for: Cuproptosis-related lncRNA SNHG16 as a biomarker for the diagnosis and prognosis of head and neck squamous cell carcinoma
Source: PeerJ. 2023 Oct 12;11:e16197. doi: 10.7717/peerj.16197 (PMC10576967; doi:10.7717/peerj.16197)

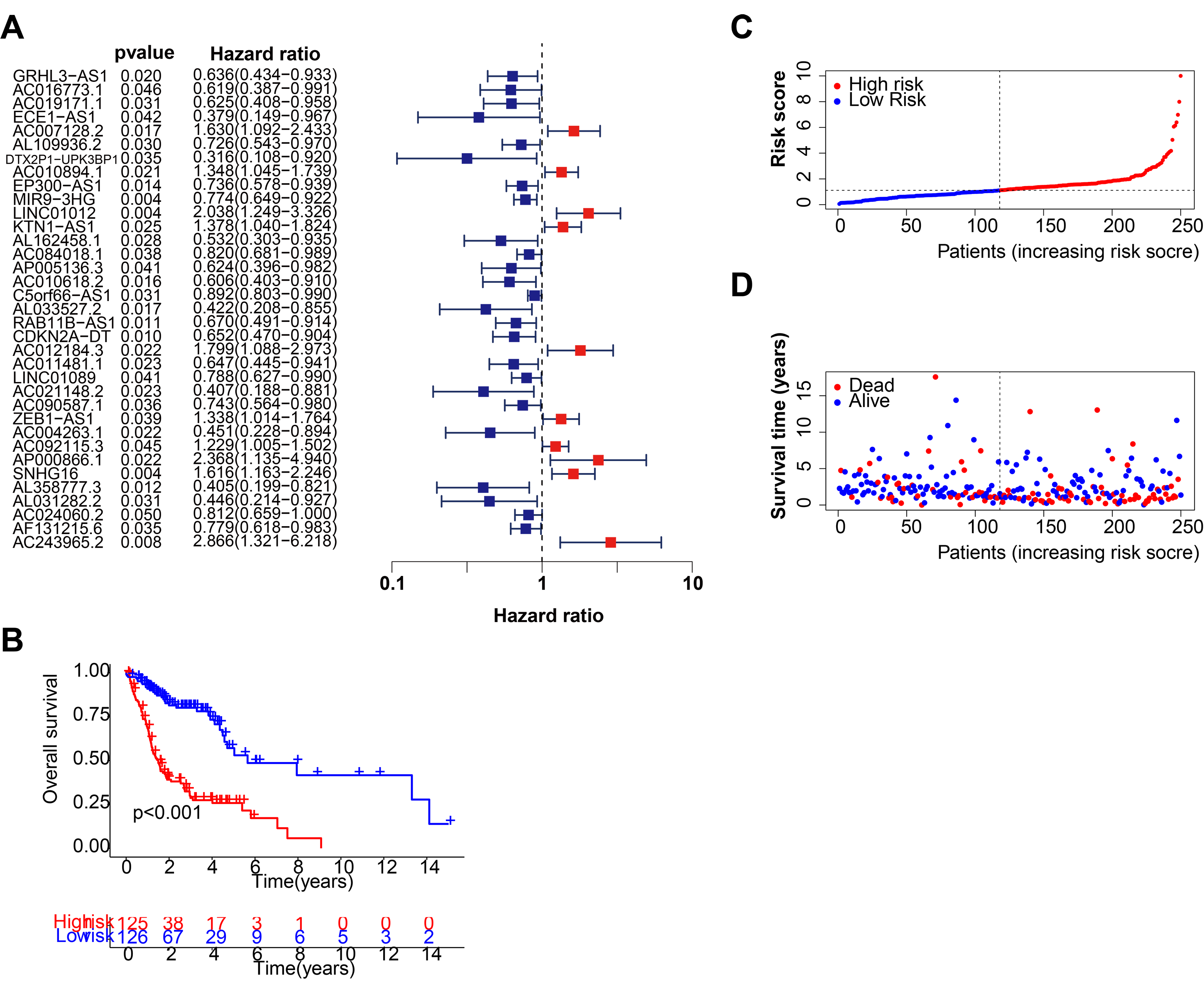

Supplement: Supplemental Information 2 — (A) Univariate Cox regression analysis was used to analyze cuproptosis-related lncRNAs that were significantly correlated with survival. (B) Total survival (OS) of patients with HNSCC in the training group. (C) Risk score distribution of patients with HNSCC with different risks (low, blue; high, red) in the testing group. (D) Dot plots showing the survival time and risk score in the testing group. [file peerj-11-16197-s002.png]
